# Supplementary material for: Post-Transcriptional Effects of miRNAs on PCSK7 Expression and Function: miR-125a-5p, miR-143-3p, and miR-409-3p as Negative Regulators
Source: Metabolites. 2022 Jun 23;12(7):588. doi: 10.3390/metabo12070588 (PMC9323720; doi:10.3390/metabo12070588)

Supplementary Table S1: Sequences of primers used in Q-PCR analysis, SOEing PCR and vector generation.

Names	Forward /Reverse	Sequences
**Expression primers**		
miR-125	F	ACGCATCCCTGAGACCCTT
mir-125	R	CAGTGCAGGGTCCGAGGTA
Stem-loop		GTCGTATCCAGTGCAGGGTCCGAGGTATTCGCACTGGATACGACTCACAG
miR-143	F	GCACGCATGAGATGAAGCAC
miR-143	R	AGTGCAGGGTCCGAGGTA
Stem-loop		GTCGTATCCAGTGCAGGGTCCGAGGTATTCGCACTGGATACGACGAGCTA
miR-320	F	ACGCAAAAAGCTGGGTTGAG
miR-320	R	AGTGCAGGGTCCGAGGTA
Stem loop		GTCGTATCCAGTGCAGGGTCCGAGGTATTCGCACTGGATACG ACTCGCCC
miR-409-3p	F	GGGAATGTTGCTCGGTG
miR-409-3p	R	CAGTGCAGGGTCCGAGGTA
Stem loop		GTCGTATCCAGTGCAGGGTCCGAGGTATTCGCACTGGAT
RNU48	F	TGATGACCCCAGGTAACTCTG
RNU48	R	AGTGCAGGGTCCGAGGTA
Stem-loop		GTCGTATCCAGTGCAGGGTCCGAGGTATTCGCACTGGATACGACGGTCAG
PCSK7	F	ACACCATCCAGGACATTGCA
PCSK7	R	GGGTCATTAGAGTTGAGGTCATAGC
B2m	F	GGGTTTCATCCATCCGACATTG
B2m	R	TGGTTCACACGGCAGGCATAC
GAPDH	F	ATGGGGAAGGTGAAGGTCG
GAPDH	R	GGGGTCATTGATGGCAACCAATA
**Cloning primers**		
PCSK7-Wild type-3'UTR	F	CCCTCGAGCCTCAGGGCCTGACAGTGT
PCSK7- Wild type 3'UTR	R	TTGCGGCCGCATTGTTTGGAAGGTTTTTATTTTGG
3'-UTR-1	F	CCTCAGGGCCTGACAGTGT
3'-UTR-1	R	CATCTGCCATCTGCTCTGC
3'-UTR-2	F	TGTTGGAGGAGAGAGCC
3'-UTR-2	R	ATTGTTTGGAAGGTTTTTATTTTG
miR-125	F	GGTCTTTCTGTCTCTGGC
miR-125	R	AGGTTTCAGTTGGTGGTC
miR-143	F	CGGAATTCCTAACACCCCTTCTCCTGGC
miR-143	R	CGGGATCCTTCCCCAGCATCACAAGTGG
miR-409	F	GTCTTCTGCAAGCACAGCC
miR-409	R	CAATTACTTTCCAACTGGTCGAC
miR-320a	F	CGGAATTCGGGGCGGAAGTGACGTTAG
miR-320a	R	CGGGATCCACCCTGATCTTGGCGCCT
**SOEing primers**		
125-1-mut a F	F	CCCTCGAGTGGGACAGGCTCTTCTTT
125-2-mut b1	R	AGATAGCTGGCCTCCGGCGGAAGACAGGGTGGTGGCAG
125-2-mut b2	F	CTGCCACCACCCTGTCTTCCGCCGGAGGCCAGCTATCT
143-1-mut a	R	CTAGGCAGCTGGCTTGGCAGGCCAAACCAAAGGGGGG
143-1-mut a	F2	CCCCCCTTTGGTTTGGCCTGCCAAGCCAGCTGCCTAG
143-2-mut b	F2	GTTGAGAAGGTGCCTGCCAAAACATCACTGGGGTCACTT
143-2-mut b	R1	AAGTGACCCCAGTGATGTTTTGGCAGGCACCTTCTCAAC
143-mut	R1	AAGTGACCCCAGTGATGTTTAGGCCAAACCAAAGGGGGG
143 mut	F2	CCCCCCTTTGGTTTGGCCTAAACATCACTGGGGTCACTT
mut 409	R	TTGCGGCCGCACCAACTAGCTCAGAAGAGGG

Supplementary Figure S1: Functional analyses of miRNAs. **A)** Expression levels of *PCSK7* after miR-125a-5p and miR-320a-3p overexpression in HepG2 cells compared with their mock vector counterpart. The expression level of *PCSK7* mRNA in HepG2 cells was considerably downregulated by miR-125a-5p (P=0.001) whilst miR-320a-3p (P=0.7830) could not downregulate the expression of *PCSK7* in HepG2 cells. **B)** Luciferase assay analysis of cells co-transfected with the miR-overexpressing vector and wildtype 3'-UTR, 3'-UTR-1 (distal) and 3'-UTR-2 –*PCSK7* psiCHECK-2 vectors in the HEK293T and HepG2 cells. The results revealed miR-125a-5p could significantly target all three constructs; wildtype 3'-UTR (P <0.0001, P <0.0001), 3'-UTR-1 (distal, P=0.0043, p=0.0247) and 3'-UTR-2 (proximal, P <0.0001, P <0.0001) which the latter ones carried the one target site (*See* Figure 1) of miR-125a-5p in the 3′-UTR in both HEK293T and HepG2 cell lines.

A)

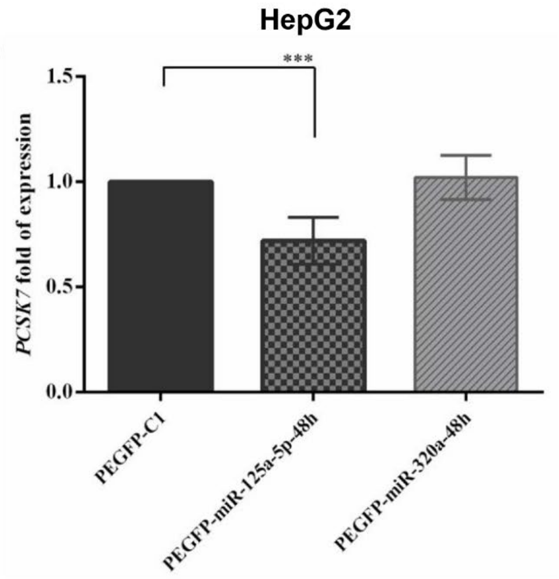

B) miR-125a-5p

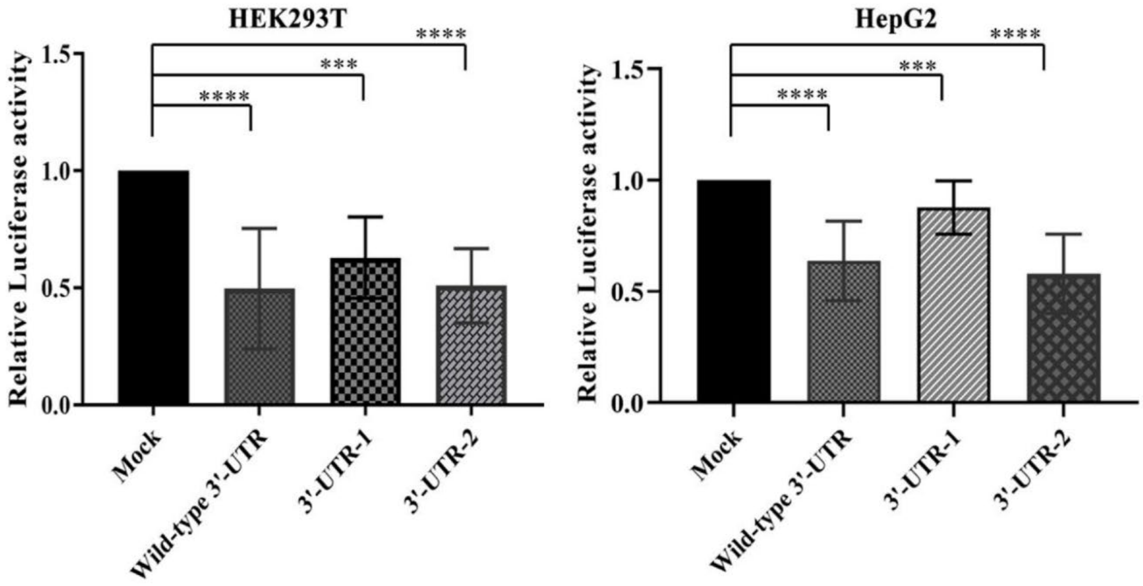

Supplementary Figure S2: Luciferase assays following co-transfection of the mixture of all three miRNAs overexpression vectors along with the wild type 3′-UTR, 3′-UTR-1 and 3′-UTR-2 parts of *PCSK7* in HEK293T cells. The relative luciferase activity did not change significantly, comparing to the effect of each microRNAs (p>0.05).

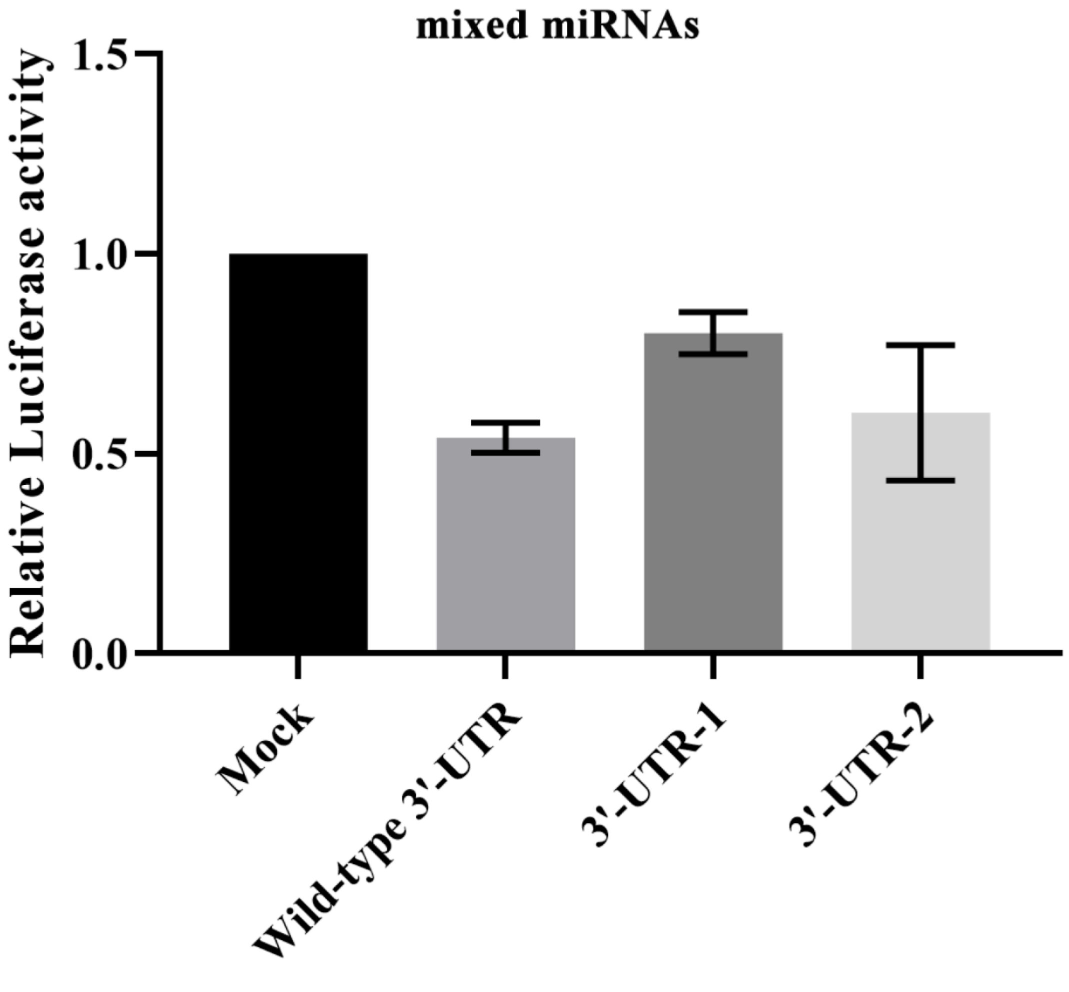

Supplementary Figure S3: Effect of miRNAs on PC7 expression. Western blot (**A**) and quantitative (**B**) analyses of the protein expression of *PCSK7* following the co-transfection of the PC7-overexpressing vector, along with different ratios of the miR-125a-5p-overexpressing vectors (1:2[2x], 1:3[3x], and 1:5[5x]) in Huh7 cells. The expression of PC7 protein decreased significantly ($p<0.0001$) in all three ratios of the miR-125a-5p-overexpressing plasmid, whereas there was no significant difference concerning the reductions of PC7 in the different amounts of miR-125a-5p. The quantitative data represent averages of 3 independent experiments.

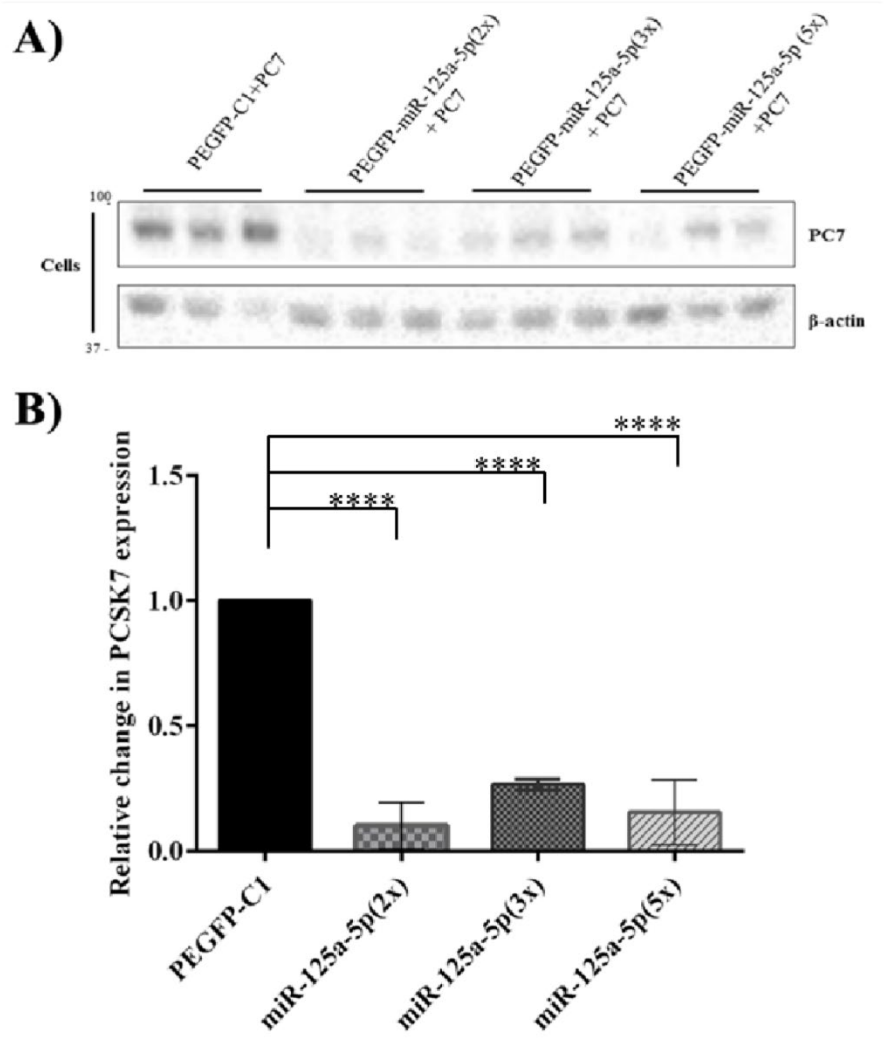

Supplementary Figure S4: Loss of functional PC7 activity on human TfR1 by miRNAs. Western blot (A) and quantitative B) analyses of the cellular expression of human TfR1 after the co-transfection of the miR-125a-5p and TfR1-overexpressing vectors in Huh7 cells. No direct effect ($P>0.05$) by miR-125a-5p on TfR1 expression was observed (orderly, 0.6974, 0.6974, 0.6806). Here, miR-125a-5p was overexpressed at different plasmid DNA ratios of PCSK7: miRNA 1:2 (2x), 1:3 (3x), and 1:5 (5x). X stands for vector DNA fold for miR-125a-5p compared to vector expressing TfR1. The quantitative data represent averages of 3 independent experiments.

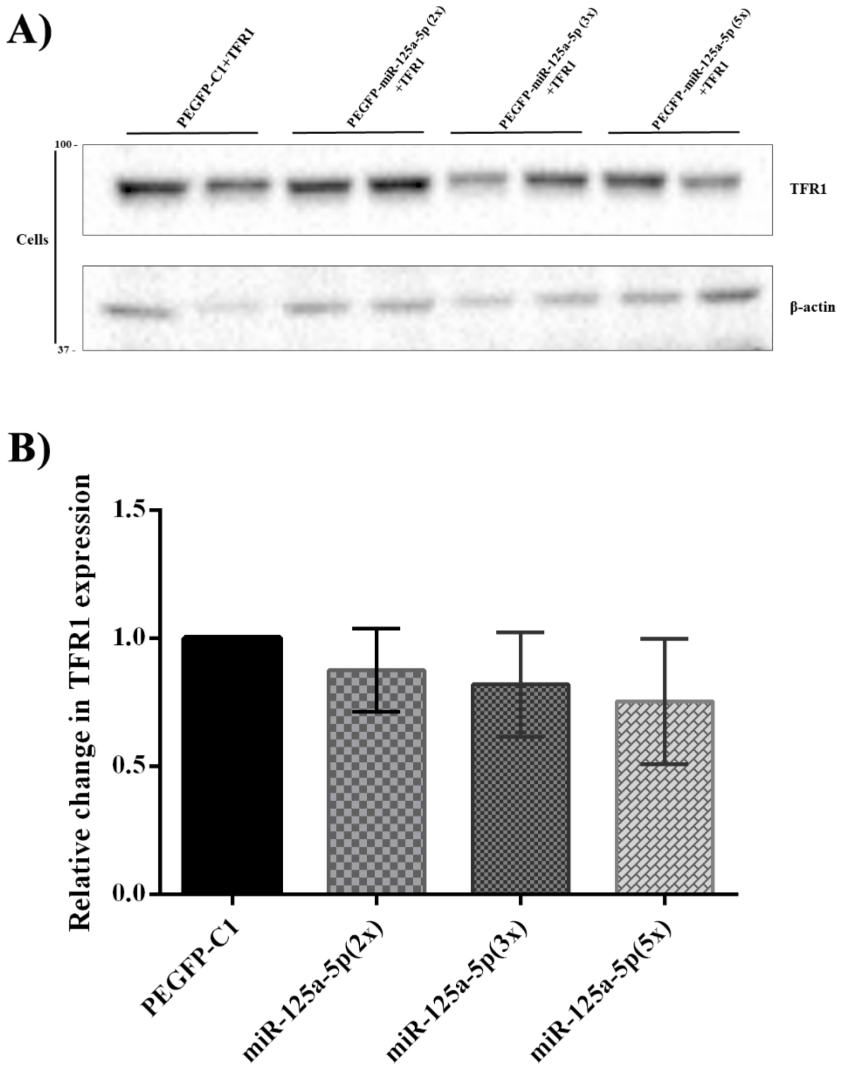

Supplement: Supplementary file 1 [file metabolites-12-00588-s001.zip › metabolites-1784624-supplementary.pdf]
